# Participation frequency in physical education classes and physical activity and sitting time in Brazilian adolescents

**DOI:** 10.1371/journal.pone.0213785

**Published:** 2019-03-13

**Authors:** Diego Augusto Santos Silva, Jean-Philippe Chaput, Mark S. Tremblay

**Affiliations:** 1 Research Center in Kinanthropometry and Human Performance, Federal University of Santa Catarina, Florianopolis, Brazil; 2 Healthy Active Living and Obesity Research Group, Children’s Hospital of Eastern Ontario Research Institute, Ottawa, Canada; Federal University of Pelotas, BRAZIL

## Abstract

**Introduction:**

To examine the association between participation frequency per week in physical education (PE) classes and physical activity (PA) and sitting time levels in adolescents according to the economic development level of the region of residence.

**Methods:**

A cross-sectional study with a sample representative of Brazil was carried out with 12,220 students aged 11–19 years. Participation frequency per week in PE classes, moderate-to-vigorous PA (MVPA), PA during PE classes, active commuting, PA outside of school hours, total accumulated PA, time sitting in front of the TV and total sitting time were assessed by using a self-administered questionnaire.

**Results:**

Adolescents who reported having PE classes were more likely to meet MVPA recommendations (1–2 PE class/week–OR: 1.3, 95%CI: 1.1–1.5; ≥3 PE class/week–OR: 2.0, 95%CI: 1.7–2.5), spent more time in PA outside of school hours (1–2 PE class/week–OR: 1.6, 95%CI: 1.4–1.9; ≥3 PE class/week–OR: 2.0, 95%CI: 1.5–2.6), and accumulated more PA (1–2 PE class/week–OR: 1.9, 95%CI: 1.6–2.2; ≥3 PE class/week–OR: 6.0, 95%CI: 4.0–8.9) than students who reported not taking PE classes. Boys from regions with higher Human Development Index (HDI) who took ≥3 PE classes/week were more likely to have higher levels of active commuting (OR: 1.4, 95%CI: 1.1–1.9) and less likely of getting in front of TV (OR: 0.7, 95%CI: 0.5–0.9). Adolescents from regions with higher HDI were more likely to have more time spent in PA during PE classes (Male–OR: 2.7, 95%CI: 2.4–3.1; Female–OR = 3.2, 95%CI: 2.8–3.7).

**Conclusions:**

Having PE classes is associated with a higher level of PA in both sexes and in both regions and lower level of sitting time in boys from regions with higher HDI.

## Introduction

Among the various strategies aimed at addressing low PA levels and high sitting time in children and adolescents, Physical Education (PE) classes in the school environment stands out [1]. Studies have shown that adolescents who have higher frequency of participation in PE classes are more likely to spend more time in moderate- to-vigorous-intensity physical activity (MVPA) and less time sitting throughout the day [2], to have better academic performance [3], greater social interactions [4] and lower cardiovascular risk [5] compared to peers who do not attend PE classes. These findings highlight the importance of PE classes as a means of improving and maintaining the health of children and adolescents.

A recent research [6] involving school-aged children from 12 countries has shown that children from low/middle-income countries who took PE classes 1–2 times/week were more likely to present better indicators of PA and shorter time in sedentary behavior in- and out-of-school than children who did not take PE classes. In high-income countries, these results were more evident in boys and with participation frequency in ≥3 PE classes/week. These differences in results between countries of different income levels can suggest that in the same country there are differences between regions with different levels of development.

The minimum number of PE classes per week suggested for children and adolescents varies among countries. For example, the minimum suggested is two classes per week in Denmark [5], while in Canada [7] and the United States [1] this number varies according to the different provinces or states, where some of them suggest one class per week and others suggest two or three classes per week. In Brazil, there are cities that adopt one class per week while others propose more classes per week [8]. However, research has shown that there are schools in Brazil that do not offer PE classes [9]. This discrepancy in the number of classes offered in the same country is an indication that PE in school still needs more standardization and regulation. One way to draw attention to this issue is to investigate the relationship between frequency participation in PE and time spent in PA and sitting time throughout the day.

Brazil is a country with socioeconomic discrepancies, in which more developed regions present better infrastructure for the practice of physical activities outside the school environment, e.g., in parks and environments with leisure areas, than less developed regions [10]. This inequality also occurs in the school environment where in more developed regions schools have more places for physical activities and sports, such as courts, swimming pools, and athletics tracks than schools located in less developed regions [10]. Investigating the relationship between involvement in PE classes and PA level and sitting time in different Brazilian regions can subsidize the discussion of socioeconomic inequality and the reflection of this in the PA promotion in children and youth.

The rationality of the relation between higher frequency in PE classes/week with higher level of PA and shorter time in the seated position is based on the theory that the practice of PA, at any intensity, causes changes at the level of the brain that stimulate vigor and more movement throughout the day [11]. Thus, it can be inferred that children who engage in PE classes will tend to be more physically active throughout the day. A recent published study reinforced this theory [6]. On the other hand, there is another theory that suggests that there is an energy expenditure threshold for children, once reached, the rest of the daily time is compensated with little or no PA [12, 13]. Here, it can be inferred that participation in PE classes helps to reach this threshold of energy expenditure and, on PE days, the PA time in the rest of the day is decreased. The authors of the present study believe that the evidences of the first theory are stronger, especially in children and young.

This study aims to examine the association between frequency of PE classes per week in PE classes and PA and sitting time levels in Brazilian high school students according to the economic development level of the region of residence. We hypothesized that a higher frequency of participation in PE classes would be associated with higher PA levels and lower sitting time in Brazilian high school students, and, due to the socioeconomic discrepancies between the regions of Brazil, these associations would be more evident in more developed regions.

## Materials and methods

### Study design

Data collected for the 2015 National Adolescent School-based Health Survey (PeNSE), a cross-sectional study, which was conducted between April and September 2015, were used in the present study. The aim of PeNSE 2015 was to assess the risk and protective factors for the health of students enrolled in public and private schools throughout Brazil [10,14].

This study used information from a sample called Number Two of the PeNSE survey 2015 [10,14]. This sample is nationally representative of Brazil and included students who were enrolled from the 6^th^ grade of elementary school to the 3^rd^ grade of high school (participants were aged from 11 to 19 years). The geographic strata used were the five regions of Brazil (Mid-Western, Northeastern, Northern, Southeastern, and Southern). The primary sampling unit was the regions, the secondary unit was the schools and the tertiary unit was the classes. Sample schools were selected from the national register of all schools in Brazil. The selection of schools based on the sample size required for each stratum was made in a random and proportional way for each school grade. Sample selection was carried out by conglomerates, selected schools in each of the regions, and, in these schools, classes in which all the students were selected and asked to answer the research questionnaires. The research was approved by the National Ethics Research Committee of Brazil and by the National Health Council of Brazil (Opinion No. 1,006,467, 2015). Informed consent was obtained from parents/legal guardians, and students assent was also obtained as required by local ethics review boards.

### Participants

Sample size was calculated for each stratum and considered sampling error of 3%, prevalence of 50%, with a confidence level of 95% and an average effect of the sampling plan of 3. These parameters allowed reaching a required sample size of 19,558 students. To reach this amount, 371 schools were visited, which resulted in 653 classes. In total, 16,608 adolescents were present at the school on the day of data collection. The other 2,950 were enrolled but did not attend school. Of the 16,608 adolescents attending schools, 16,556 accepted to participate in the survey. Further details on the sampling process can be found elsewhere [10].

Of the 16,556 students who accepted to participate in the study, 12,220 adolescents comprised the study sample. Participants who did not answer questions about maternal schooling (n = 4,202), overall PA level (n = 53), out-of-school PA (n = 23), PA time in PE class (n = 24), total sitting time (n = 22), sitting time in front of the TV (n = 11) and active commuting (n = 1) were excluded. Children who were excluded for missing data did not significantly differ in their descriptive characteristics (region of residence and age) from those who were included in the present analysis.

Data collection staff had a background on the project, and were subsequently trained by research staff. Data collection procedures followed the published [10,14], which provided standardized procedures to collect data across the survey.

### Physical activity and sitting time measures

PA during PE classes, active commuting, PA outside of school hours, total accumulated PA (Total PA = PA during PE classes + active commuting + PA outside of school hours), MVPA time, time sitting in front of the TV, and total sitting time were assessed by using a self-administered questionnaire [10]. The PeNSE questionnaire [10,14] was designed by using health indicators used in the Global School-based Student Health Survey (GSHS), developed by the World Health Organization [15]. PA indicators and sitting time were validated in a sample of Brazilian adolescents and showed high agreement with the measures of a 24-hour recall [16].

For the PA indicators, questionnaire items referred to the last seven days. Adolescents were instructed to consider a normal week of class, without holidays or vacations. For sitting time, students were instructed to consider a typical weekday, disregarding Saturday, Sunday, holiday, or sitting time at school [10,14].

For PA during PE classes, students were asked how long they performed PA or sport during PE classes. They were also asked how many days per week they took PE classes. The information collected allowed obtaining the weekly amount in minutes of this activity. For active commuting, students were asked if they went to school on foot or by bicycle and how long the journey lasted. The same questions were asked for the return from the school. In the present study, the total time of active commuting was considered, adding time to school and returning from school. For PA outside of school hours, adolescents were asked how many days, apart from PE classes, they practiced some PA or sport. Subsequently, they were asked how long this activity usually lasted. The information was considered to obtain the total time of PA outside of school hours. Total accumulated PA was calculated as described above (Total PA = PA during PE classes + active commuting + PA outside of school hours). In addition, we created a new variable for total accumulated PA without the minutes of PA in PE classes (Total accumulated PA without the minutes of PA in PE classes = Active commuting + PA outside of school hours). MVPA time was investigated by a single question in which adolescents were questioned for how many days they did at least 60 minutes of MVPA. This information was used to estimate how many minutes/week they spent in MVPA [10,14].

For sitting time indicators, adolescents were asked how many hours/day they watched TV and what was their total sitting time, considering the time they spent in front of the TV, using a computer, playing video games, talking to friends, or doing any other activities in the sitting position [10,14].

Adolescents were classified as meeting the MVPA recommendation of ≥60 min/day, or not [17,18]. They were also classified according to the time spent in front of the TV, where it is recommended that the time should not exceed 2 h/day [17]. In addition, each PA indicator (min/week) and the overall sitting time (min/day) were categorized according to the tertile distribution for boys and girls. PA and sitting time indicators did not present a normal distribution, so we chose to carry out analyzes with categorical variables. We chose this classification because there are no specific recommendations for each PA indicator and the overall sitting time. Sensitivity analyses conducted by using other cutoff points for PA and sitting time presented similar patterns of associations; therefore, only tertiles are presented in the present paper.

### Frequency of participation in PE classes

For this measure, questionnaire item referred to the last seven days. Adolescents were instructed to consider a normal week of class, without holidays or vacations. Adolescents were asked how many days they had PE classes in school. The answer options were “no day in the last seven days,” or “1,” “2,” “3,” “4,” “5,” or “more than 5 days.” The responses were classified as “0 days/week,” “1–2 days/week,” and “≥3 days/week” because the results of associations between frequency of participation in PE classes and PA/sitting time for 1 and 2 days/week; and 3, 4, and 5 or more days/week were similar.

### Geographic regions of residence

The five geographic regions of Brazil were grouped according to the Human Development Index (HDI) [19] and classified into regions with higher HDI (Mid-Western = 0.753, Southeast = 0.753, Southern = 0.756) or in regions with lower HDI (Northern = 0.683, Northeastern = 0.659). This form of classification is used in studies of social inequalities in Brazil [20].

### Control variables

Sex, age, maternal education, and body mass index (BMI) z-scores were included as covariates. These variables were chosen because of their association with the dependent and independent variables in previous studies [9]. Sex was self-reported, and age was collected in complete years and classified into ≥11 ≤13; ≥14 ≤16; and ≥17 ≤19 years. Maternal schooling was reported by participants and classified into ≤8; >8 ≤12; and >12 years of study. Body mass was measured with a portable electronic scale after all outer clothing, heavy pocket items, and shoes were removed. Body height was measured without shoes by using a portable stadiometer, with participant’s head in the Frankfort plane, and after a deep inspiration. Each measurement was repeated, and the average was used for analysis (a third measurement was obtained if the first two measurements were greater than 0.5 kg or 0.5 cm, respectively, and the average of the two closest measurements was used for analysis) [10]. BMI (kg/m2) was calculated, and BMI z-scores were computed by using age- and sex-specific reference data from the World Health Organization [21].

### Statistical analysis

Descriptive statistics (mean, standard deviation, absolute frequency, relative frequency) on the sample characteristics were presented for each geographic region. The chi-square test was used to compare the proportions of PA and sitting time index of adolescents according to participation frequency in PE classes. A multi-level logistic mixed regression model was used where the dependent variable was meeting MVPA recommendations and time sitting in front of the TV [17,18]. Multi-level polytomous mixed logistic regression models were used [22], where the dependent variables were the different PA indicators (PA during PE classes, active commuting, PA outside of school hours, accumulated PA) and total sitting time (sex-specific reference categories = 1^st^ (lowest) tertile for PA indicators and 3^rd^ (highest) tertile for total sitting time). Odds ratios and 95% confidence intervals were estimated. Geographic region was considered to have fixed effects and schools located within study regions were viewed as having random effects. Age, maternal education, BMI z-scores, and meeting the MVPA recommendations (with models containing TV time or total sitting time as dependent variable) and total sitting time (with models containing PA indices as dependent variables) were included as covariates. Analyses were performed for the whole sample, stratified by sex, and by region-level classification of economic development. The analysis were stratified by sex because there was interaction between sex and frequency of participation in PE classes/week. Statistical analyses were performed by using Stata 13.0 (STATA Corp. College Station, Texas, USA). The statistical significance level was p < 0.05. All analyses considered the design effect and the sampling weight. The person conducting the analyzes was blinded to the research objectives and sample conditions.

## Results

**Fig 1:**
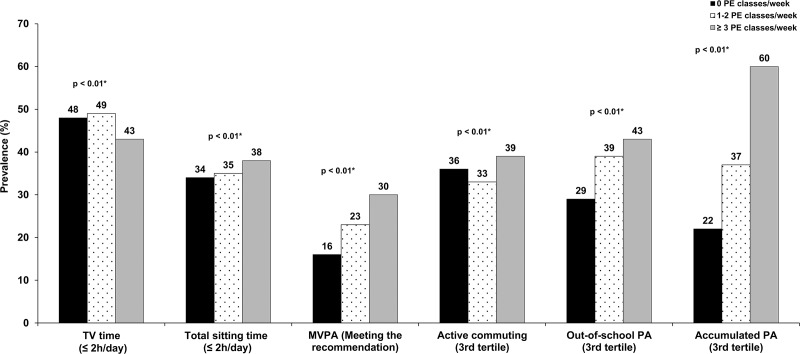
Comparison of the proportions of physical activity indicators and sitting time among adolescents according to participation frequency in physical education classes per week (n = 12,220); *p< 0.01 (chi-square test). PE: physical education; MVPA: moderate-to-vigorous physical activity; PA: physical activity.

Table 1 shows the sample distribution according to the participation frequency in PE classes and economic development of Brazilian regions. Students who took three or more PE classes/week had higher prevalence of meeting the MVPA recommendation (30.0%), were in the highest tertile of time spent in active commuting (39.0%), PA outside of school hours (43.0%), total accumulated PA (60.0%) and spent less time in the sitting position (38.0%) than students who did not take any PE classes at school (Fig 1).

**Table 1:** Descriptive characteristics of the sample (n = 12,220).

	0 PE classes/week (n = 3,969)			1–2 PE classes/week (n = 7,163)			≥ 3 PE classes/week (n = 1,088)		
	Full sample	Regions with lower HDI	Regions with higher HDI	Full sample	Regions with lower HDI	Regions with higher HDI	Full sample	Regions with lower HDI	Regions with higher HDI
	n (%)	n (%)	n (%)	n (%)	n (%)	n (%)	n (%)	n (%)	n (%)
**Sex**									
Male	1,721 (43.4)	939 (43.2)	782 (43.6)	3,717 (51.9)	1,285 (52.7)	2,432 (51.5)	596 (54.8)	199 (58.4)	397 (53.1)
Female	2,248 (56.6)	1,236 (56.8)	1,012 (56.4)	3,446 (48.1)	1,155 (47.3)	2,291 (48.5)	492 (45.2)	142 (41.6)	350 (46.9)
**Maternal education (years)**									
≤8	1,506 (37.9)	871 (40.0)	635 (35.4)	2,422 (33.8)	839 (34.4)	1,583 (33.5)	469 (43.1)	157 (46.0)	312 (41.7)
>8 ≤12	1,339 (33.7)	748 (34.4)	591 (32.9)	2,194 (30.6)	745 (30.5)	1,449 (30.7)	290 (26.7)	90 (26.4)	200 (26.8)
>12	1,124 (28.4)	556 (25.6)	568 (31.7)	2,547 (35.6)	856 (35.1)	1,691 (35.8)	329 (30.2)	94 (27.6)	235 (31.5)
**BMI z-scores**									
Low weight	116 (2.9)	65 (3.0)	51 (2.8)	226 (3.2)	94 (3.9)	132 (2.8)	31 (2.8)	09 (2.6)	22 (2.9)
Eutrophic	2,842 (71.6)	1,615 (74.3)	1,227 (68.4)	4,928 (68.8)	1,717 (70.4)	3,211 (68.0)	727 (66.8)	243 (71.3)	484 (64.8)
Overweight	708 (17.8)	351 (16.1)	357 (19.9)	1,327 (18.5)	412 (16.9)	915 (19.4)	222 (20.5)	58 (17.0)	164 (22.0)
Obesity	303 (7.7)	144 (6.6)	159 (8.9)	682 (9.5)	217 (8.8)	465 (9.8)	108 (9.9)	31 (9.1)	77 (10.3)
**MVPA (meeting the recommendation)**									
No	3,354 (84.5)	1,836 (84.4)	1,518 (84.6)	5,552 (77.5)	1,908 (78.2)	3,644 (77.2)	763 (70.1)	239 (70.1)	524 (70.1)
Yes	615 (15.5)	339 (15.6)	276 (15.4)	1,611 (22.5)	532 (21.8)	1,079 (22.8)	325 (29.9)	102 (29.9)	223 (29.9)
**TV Time (h/day)**									
≤ 2h	1,918 (48.3)	1,013 (46.6)	905 (50.4)	3,480 (48.6)	1,180 (48.4)	2,300 (48.7)	471 (43.3)	162 (47.5)	309 (41.4)
> 2h	2,051 (51.7)	1,162 (53.4)	889 (49.6)	3,683 (51.4)	1,260 (51.6)	2,423 (51.3)	617 (56.7)	179 (52.5)	438 (58.6)
**Total sitting time**									
≤ 2h/day	1,359 (34.3)	818 (37.6)	541 (30.2)	2,508 (35.0)	905 (37.0)	1,603 (33.9)	413 (38.0)	147 (43.1)	266 (35.6)
> 2h/day ≤ 4h/day	989 (24.9)	547 (25.1)	442 (24.6)	1,911 (26.7)	665 (27.3)	1,246 (26.4)	256 (23.5)	65 (19.1)	191 (25.6)
> 4h/day	1,621 (40.8)	810 (37.3)	811 (45.2)	2,744 (38.3)	870 (35.7)	1,874 (39.7)	419 (38.5)	129 (37.8)	290 (38.8)
		**Mean (S.D.)**			**Mean (S.D.)**			**Mean (S.D.)**	
**Age (years)**	15.2 (2.0)	15.1 (2.1)	15.2 (1.8)	14.1 (2.0)	14.0 (2.0)	14.1 (2.1)	13.3 (1.9)	13.4 (2.1)	13.2 (1.8)
**PA in PE classes (min/week)**	0.0 (0.0)	0.0 (0.0)	0.0 (0.0)	57.1 (34.4)	50.6 (32.8)	60.4 (34.8)	160.8 (104.5)	177.6 (119.1)	153.1 (96.3)
**Active commuting (min/week)**	74.1 (109.5)	76.1 (10.8.7)	71.5 (110.4)	71.2 (108.4)	71.9 (110.2)	70.8 (107.6)	94.5 (139.8)	111.8 (147.0)	86.6 (135.7)
**Out-of-school PA (min/week)**	93.4 (138.1)	92.3 (138.4)	94.7 (137.7)	132.3 (147.3)	125.2 (144.8)	135.9 (148.5)	148.0 (150.8)	148.1 (151.6)	148.0 (150.5)
Accumulated PA without PA in PE class (min/week)*	167.5 (177.7)	168.4 (176.7)	166.2 (178.9)	203.4 (186.8)	197.1 (181.2)	206.7 (189.5)	242.5 (212.5)	259.9 (210.4)	234.5 (213.2)
**Accumulated PA (min/week)†**	167.5 (177.7)	168.4 (176.7)	166.2 (178.9)	260.6 (195.8)	247.7 (189.4)	267.1 (198.8)	403.3 (268.9)	437.5 (278.4)	387.7 (263.3)

HDI: human development index; PE: physical education; PA: physical activity; BMI: body mass index; MVPA: moderate- to vigorous-intensity physical activity

*Total accumulated PA = Active commuting + PA outside of school hours

†Total accumulated PA = PA during PE classes + active commuting + PA outside of school hours.

**Fig 2:**
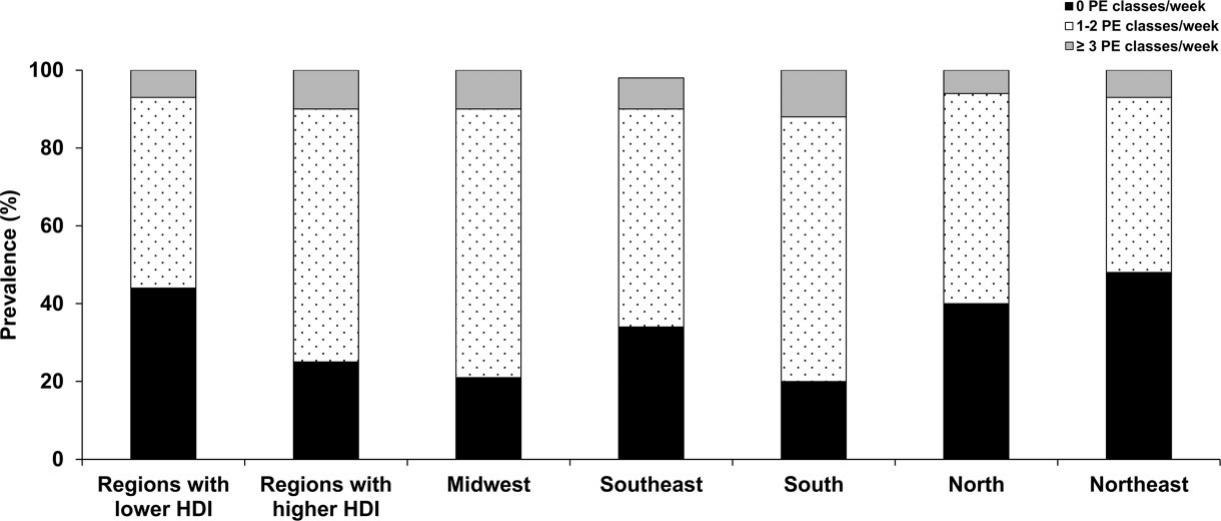
Distribution of participation frequency in physical education classes per week by study region (n = 12,220). HDI: Human Development Index; PE: physical education.

The less developed regions of Brazil (North and Northeast) had a higher proportion of adolescents who did not take any PE classes at school than regions with higher HDI. The more developed regions of Brazil (Midwest, Southeast and South) presented a higher proportion of adolescents who took 1–2 PE classes/week and 3 or more PE classes/week than regions with lower HDI. Fig 2 shows the distribution of participation in PE classes according to the different Brazilian regions.

Adolescents of both sexes living in regions with higher or lower HDI who took 1–2 PE classes/week and 3 or more PE classes/week were more likely to meet the MVPA recommendation, to spend more time in PA outside of school hours and total accumulated PA than adolescents who did not take any PE classes/week (Table 2). For boys living in regions with higher HDI who took 1–2 PE classes/week and 3 or more PE classes/week were more likely to spend more time in active commuting and spent less time in the sitting position than boys who did not take any PE classes/week (Table 2).

**Table 2:** Association between participation frequency in physical education classes and indicators of physical activity and sitting time according to sex and income level of the Brazilian regions (n = 12,220).

	Male (n = 6,034)											
	All the sample				Regions with lower HDI				Regions with higher HDI			
	1–2 PE classes/week		≥ 3 PE classes/week		1–2 PE classes/week		≥ 3 PE classes/week		1–2 PE classes/week		≥ 3 PE classes/week	
	OR	(95%CI)	OR	(95%CI)	OR	(95%CI)	OR	(95%CI)	OR	(95%CI)	OR	(95%CI)
**MVPA (meeting the recommendation)**												
Yes	1.3	(1.1–1.5)**	2.0	(1.7–2.5)**	1.2	(1.1–1.5)*	1.9	(1.3–2.6)**	1.4	(1.2–1.7)**	2.2	(1.6–2.9)**
**Active commuting (tertile–min/day)**												
2^nd^ (> 0.0 < 70.0)	1.1	(0.9–1.3)	1.2	(0.9–1.6)	0.9	(0.7–1.1)	1.2	(0.8–1.8)	1.3	(1.1–1.6)*	1.3	(0.9–1.8)
3^rd^ (≥ 70.0)	1.1	(0.8–1.2)	1.4	(1.1–1.8)**	0.8	(0.7–1.1)	1.7	(1.1–2.4)**	1.1	(0.9–1.3)	1.4	(1.1–1.9)*
**Out-of-school PA (tertile–min/day)**												
2^nd^ (> 29.9 < 195.0)	2.1	(1.8–2.4)**	2.7	(2.0–3.4)**	1.8	(1.4–2.3)**	2.8	(1.8–4.2)**	2.2	(1.8–2.7)**	2.5	(1.8–3.6)**
3^rd^ (≥ 195.0)	1.6	(1.4–1.9)**	2.0	(1.5–2.6)**	1.4	(1.1–1.8)**	2.1	(1.3–3.4)**	1.7	(1.3–2.1)**	1.8	(1.3–2.6)**
**Accumulated PA without PA in PE class (tertile–min/day)†**												
2^nd^ (> 104.9 < 295.0)	1.1	(0.7–1.6)	1.0	(0.6–1.6)	1.3	(0.9–1.8)	1.6	(0.7–3.6)	0.8	(0.5–1.4)	0.6	(0.4–0.9)*
3^rd^ (≥ 295.0)	1.2	(0.9–1.5)	1.6	(1.1–2.4)*	1.3	(1.1–1.7)*	2.1	(1.2–4.0)*	0.9	(0.7–1.3)	1.1	(0.7–1.9)
**Accumulated PA (tertile–min/day)‡**												
2^nd^ (> 144.9 < 350.0)	1.4	(1.1–1.8)*	2.2	(1.5–3.2)**	1.4	(1.1–2.0)**	2.8	(1.3–5.6)**	1.3	(0.8–1.8)	1.7	(1.2–2.5)**
3^rd^ (≥ 350.0)	1.9	(1.6–2.2)**	6.0	(4.0–8.9)**	1.8	(1.4–2.2)**	7.3	(3.4–15.7)**	1.9	(1.5–2.2)**	5.0	(3.1–7.7)**
**TV Time (h/day)**												
≤ 2h	1.0	(0.8–1.2)	0.8	(0.7–1.1)	1.2	(0.9–1.4)	1.2	(0.8–1.7)	0.9	(0.7–1.1)	0.7	(0.5–0.9)*
**Total sitting time (h/day)**												
> 2h ≤ 4h	1.3	(1.1–1.6)*	1.1	(0.5–2.4)	1.2	(0.9–1.6)	0.7	(0.5–1.2)	1.4	(1.1–1.9)*	1.6	(1.1–2.4)*
≤ 2h	0.9	(0.5–1.4)	1.0	(0.6–1.5)	1.0	(0.8–1.3)	0.9	(0.7–1.4)	1.1	(0.7–1.7)	1.2	(0.9–1.6)
	**Female (n = 6,186)**											
**MVPA (meeting the recommendation)**												
Yes	1.5	(1.3–1.8)**	2.0	(1.5–2.6)**	1.6	(1.2–2.1)**	2.5	(1.6–3.8)**	1.4	(1.1–1.8)*	1.7	(1.2–2.4)**
**Active commuting (tertile–min/day)**												
2^nd^ (> 0.0 < 60.0)	1.1	(0.9–1.2)	1.1	(0.8–1.4)	0.9	(0.7–1.2)	1.0	(0.6–1.7)	1.1	(0.9–1.3)	1.1	(0.8–1.6)
3^rd^ (≥ 60.0)	0.9	(0.8–1.1)	1.1	(0.9–1.4)	0.8	(0.7–1.1)	1.2	(0.8–1.8)	1.1	(0.9–1.3)	1.2	(0.9–1.6)
**Out-of-school PA (tertile–min/day)**												
2^nd^ (> 0.0 < 75.0)	2.0	(1.7–2.3)**	2.2	(1.6–2.8)**	1.9	(1.5–2.4)**	1.9	(1.2–3.1)**	2.1	(1.7–2.6)**	2.5	(1.7–3.4)**
3^rd^ (≥ 75.0)	2.1	(1.8–2.4)**	2.3	(1.8–3.0)**	2.0	(1.6–2.5)**	2.2	(1.4–3.5)**	2.2	(1.8–2.7)**	2.4	(1.8–3.4)**
**Accumulated PA without PA in PE class (tertile–min/day)†**												
2^nd^ (> 49.9 < 195.0)	1.2	(1.1–1.4)*	1.1	(0.7–1.6)	1.1	(0.8–1.4)	1.0	(0.5–1.8)	1.3	(0.9–1.8)	1.2	(0.6–2.0)
3^rd^ (≥ 195.0)	1.5	(1.2–1.8)**	1.8	(1.3–2.6)**	1.6	(1.1–2.2)*	1.9	(1.2–3.0)**	1.4	(1.1–1.7)**	1.7	(1.1–2.7)*
**Accumulated PA (tertile–min/day)‡**												
2^nd^ (> 79.9 < 235.0)	2.0	(1.7–2.5)**	3.0	(1.8–4.8)**	1.6	(1.3–2.1)**	3.1	(1.5–6.2)**	2.4	(1.6–3.4)**	2.9	(1.4–5.8)**
3^rd^ (≥ 235.0)	2.5	(2.0–3.0)**	6.6	(4.3–10.8)**	2.3	(1.6–3.2)**	7.8	(4.2–14.2)**	2.5	(2.0–3.0)**	5.6	(3.3–9.4)**
**TV Time (h/day)**												
≤ 2h	1.1	(0.9–1.2)	1.0	(0.8–1.3)	1.2	(0.9–1.4)	1.3	(0.9–2.0)	0.9	(0.8–1.2)	0.9	(0.6–1.2)
**Total sitting time (h/day)**												
> 2h ≤ 4h	0.9	(0.8–1.2)	0.8	(0.5–1.4)	0.9	(0.3–2.5)	0.6	(0.2–10.4)	1.1	(0.8–1.5)	0.9	(0.4–2.2)
≤ 2h	1.0	(0.8–1.1)	0.9	(0.7–1.3)	1.0	(0.3–3.8)	1.1	(0.1–17.0)	1.1	(0.8–1.3)	1.1	(0.9–1.3)

OR: odds ratio; CI: confidence interval; PE: physical education; PA: physical activity; MVPA: moderate-to-vigorous physical activity; HDI: human development index. Models are adjusted for age, maternal education, body mass index z-scores, meeting the MVPA recommendation (with models containing TV time and sitting time as dependent variables), and time sitting (with models containing the PA indicators as dependent variables). Reference category = 0 physical education classes/week

*p < 0.05

**p < 0.001

†Total accumulated PA = Active commuting + PA outside of school hours

‡Total accumulated PA = PA during PE classes + active commuting + PA outside of school hours.

When analyzing the time spent in PA during PE classes it was found that boys and girls living in regions with higher HDI were more likely to spend more time in PA during PE classes compared to adolescents living in regions with lower HDI (Table 3).

**Table 3:** Association between physical activity time during physical education classes and geographic region of residence according to sex (n = 8,251).

	Male (n = 6,034)				Female (n = 6,186)			
	PA in PE classes				PA in PE classes			
	2^nd^ tertile (min/week) > 14.9 < 65.0		3^rd^ tertile (min/week) ≥ 65.0		2^nd^ tertile (min/week) > 0.0 < 50.0		3^rd^ tertile (min/week) ≥ 50.0	
	OR	(95%CI)	OR	(95%CI)	OR	(95%CI)	OR	(95%CI)
**Region**								
Lower HDI	1.0		1.0		1.0		1.0	
Higher HDI	1.7	(1.6–2.0)	2.7	(2.4–3.1)**	2.2	(1.9–2.5)**	3.2	(2.8–3.7)**

OR: odds ratio; CI: confidence interval; PE: physical education; PA: physical activity; HDI: human development index. Models are adjusted for age, sex (with models containing the full sample), maternal education, body mass index z-scores, and total time sitting. Reference category = 1^st^ tertile or lower physical activity during physical education classes.

*p< 0.05

**p< 0.01.

## Discussion

This study is the first one with a representative sample of Brazil that found direct association between PE classes and higher levels of PA and less time in the sitting position among adolescents. These associations were consistent for PA in- and out-of-school. This study also adds information on the fact that adolescents from developed regions of Brazil attended more classes of PE per week than adolescents from less developed regions and, therefore, were more likely to be active throughout the day.

Brazilian adolescents who had PE classes showed greater time performing PA outside of school hours, in total accumulated PA, and in meeting the MVPA recommendation. Other studies carried out with adolescents from Denmark [23], Spain [24], and the United States [25] reported that PE classes represent an avenue that can assist in the involvement of students in PA in- and out-of-school. Thus, this study reinforces the need for PE classes to be offered to promote PA among children and adolescents.

In the most developed regions of Brazil, students spent more time in PA during PE classes. That is, even when students from less developed regions took PE classes, they did less PA than those living in more developed regions of Brazil. One of the explanations for this observation may be the fact that in the less developed regions of Brazil, schools did not have adequate infrastructure for sports and PA. Data from the 2015 Brazilian School Census [26] have shown that 65.5% of public schools in Brazil did not have sports courts. When comparing Brazilian cities, the highest proportions of schools without sports courts were found in the Northern and Northeastern regions [26], which were the regions with the lowest HDI for this study. With a lack of infrastructure, teachers have to adapt or even propose sedentary activities during most PE classes, which can negatively influence the PA levels of adolescents.

As with the present study, other investigations have reported that participation in PE classes was associated with sports and PA performed outside of school hours [2,25], which is one more indication that PE classes are important for enhancing PA among children and adolescents throughout the day. Due to the cross-sectional design of this study, it is not possible to state if students taking PE classes are those who practice sports outside the school and therefore feel more motivated and able to participate in PE classes, or whether PE classes stimulate and prepare the students to be more active throughout the day. However, both approaches have already been studied in other contexts [23,27]. Research conducted in Belgium schools found that adolescents who spent more time in out-of-school sports felt more competent for motor activities and were more motivated to participate in PE classes than those who spent less time in out-of-school activities [27]. An intervention carried out in schools in Denmark has shown that increasing weekly PE classes resulted in more physically active adolescents compared to those in the control group who did not increase the number of PE classes [23].

The present study found that boys living in regions with higher HDI who took 1–2 PE classes/week and 3 or more PE classes/week were more likely to spend more time in active commuting. For boys living in less developed regions these associations did not exist. The relationship between participation in PE classes and active commuting is not well established in the literature. This population study is the first one to investigate this relationship. But the relation between active commuting being more evident in more economically developed cities may be related to a greater sense of street safety in these cities, and for this reason, children go to school children go to school walking or cycling [28]. As this study is theoretically based on others who stated that the practice of PA, at any intensity, causes changes at the level of the brain that stimulate vigor and more movement throughout the day [6]. Thus, it can be inferred that children who engage in PE classes will tend to be more physically active throughout the day, and perhaps that is why in cities with greater sense of safety on the streets, the active commuting may be more evident in children who participate in more PE classes.

The present study found that boys living in regions with higher HDI who took 1–2 PE classes/week and 3 or more PE classes/week were more likely to spend less time sitting. For boys living in less developed regions these associations did not exist. One possible explanation for this finding may be the fact that in regions with higher HDI, fewer students reported not attending PE classes and had higher frequencies in these classes. This higher frequency and engagement in PE classes may expose students to more PA and less sedentary time [4–6]. This time sitting issue, especially for the differences between educational screen time and leisure screen time [29]. Quality TV-well-designed, age-appropriate programs with specific educational goals—can provide an additional route to early language and literacy for children. Quality programming also fosters aspects of cognitive development, including positive racial attitudes and imaginative play [29]. Early evidence suggests that interactive media, specifically applications that involve contingent responses from an adult (i.e., timely reactions to what a child says or does) can help children and youth retain taught information [29]. On the other hand, the leisure time of the children and youth cannot be spent in the seated position or in front of screens, because it impairs the motor development and brings higher risks of obesity and hypertension still in adolescence [17].

The present study found that the statistical model for total accumulated PA that considered PA during PE classes showed higher odds ratio than the statistical model for total accumulated PA that did not consider PA during PE classes. In other words, adolescents of both sexes living in regions with higher or lower HDI who took 1–2 PE classes/week and 3 or more PE classes/week were more likely to spend more time in total accumulated PA than adolescents who did not take any PE classes/week, and that if the time spent in PA during PE classes is not considered in total accumulated PA/week, these odds of being physically active decrease or are not significant. This result reinforces the importance of PE classes in promoting PA among adolescents. These results are in line with other literature results [2,6]. The population has to understand that PE classes are one of the means for young people to practice PA, which can give them a greater taste for practice and make them practice at times outside of school. Thus, PE classes should be mandatory in all school cycles, as it is a clear means of promoting PA and health.

This study has important limitations that warrant attention. First, the cross-sectional study design does not allow causal inferences to be made. Second, data on frequency participation in PE classes, PA, and sitting time were obtained through a self-report questionnaire. Although questionnaires are useful for surveys with large sample sizes due to the low operational cost [17], they do not provide the same level of accuracy as accelerometry [17]. Third, information from only one screen device (e.g., TV time) limits inferences to all other screens available to adolescents. Four, the sitting time questions showed questionable validity against a 24-h recall, which is a poor golden standard to a validation study [16]. This may explain why associations were not found between frequency of participation in PE classes and sitting time in girls.

The strengths of this study lie in its external validity, since the probabilistic sampling strategy that guaranteed the sample representativeness of Brazilian public and private school students from different regions was conducted in a thorough way and according to literature recommendations [14]. Another strength was the collection of information through the Personal Digital Assistant (PDA), which allowed the responses of students to be directly recorded in an electronic questionnaire, without interference from the interviewer, and also reduced the possibility of data entry errors. Finally, the present study is the largest of its kind to assess the relationship between participation frequency in PE classes and PA and sitting time in Brazilian adolescents.

In conclusion, adolescents who had PE classes at school had more time spent in PA outside of school hours, in accumulated PA, in MVPA, and in their chance of meeting MVPA recommendations. For adolescents living in regions with higher HDI who had PE classes at school, it was found that attending at least 1–2 PE classes per week was associated with better PA indicators in- and out-of-school and with less time spent sitting daily than adolescents living in regions with lower HDI, especially in boys. In addition, adolescents living in regions with higher HDI spent more time in PA during PE classes than those living in regions with lower HDI. Furthermore, the total accumulated PA of the adolescent during the week is influenced by the participation in PE classes, so this study highlights the importance of at least 1–2 PE classes/week in the studied age group.

## Supporting information

S1 Dataset Database of the study. PENSE_AMOSTRA2.csv.
(CSV)
